# Recent Advances of Solution-Processed Heterojunction Oxide Thin-Film Transistors

**DOI:** 10.3390/nano10050965

**Published:** 2020-05-18

**Authors:** Yanwei Li, Chun Zhao, Deliang Zhu, Peijiang Cao, Shun Han, Youming Lu, Ming Fang, Wenjun Liu, Wangying Xu

**Affiliations:** 1 Shenzhen Key Laboratory of Special Functional Materials, College of Materials Science and Engineering, Guangdong Research Center for Interfacial Engineering of Functional Materials, Shenzhen University, Shenzhen 518060, China; yanweilo@163.com (Y.L.); dlzhu@szu.edu.cn (D.Z.); pjcao@szu.edu.cn (P.C.); hsdf52690@126.com (S.H.); ymlu@szu.edu.cn (Y.L.); m.fang@szu.edu.cn (M.F.); liuwj@szu.edu.cn (W.L.); 2 Department of Electrical and Electronic Engineering, Xi’an Jiaotong-Liverpool University, Suzhou 215123, China; Chun.Zhao@xjtlu.edu.cn

**Keywords:** heterojunction, metal oxide semiconductor, thin-film transistors, solution-processed

## Abstract

Thin-film transistors (TFTs) made of metal oxide semiconductors are now increasingly used in flat-panel displays. Metal oxides are mainly fabricated via vacuum-based technologies, but solution approaches are of great interest due to the advantages of low-cost and high-throughput manufacturing. Unfortunately, solution-processed oxide TFTs suffer from relatively poor electrical performance, hindering further development. Recent studies suggest that this issue could be solved by introducing a novel heterojunction strategy. This article reviews the recent advances in solution-processed heterojunction oxide TFTs, with a specific focus on the latest developments over the past five years. Two of the most prominent advantages of heterostructure oxide TFTs are discussed, namely electrical-property modulation and mobility enhancement by forming 2D electron gas. It is expected that this review will manifest the strong potential of solution-based heterojunction oxide TFTs towards high performance and large-scale electronics.

## 1. Introduction

Today, there is a growing demand for flat-panel displays with higher resolution, larger screen sizes, better viewing, and lower power consumption, pushing traditional amorphous silicon thin-film transistor (TFT) technology to its limits [1,2,3]. TFTs made of metal oxide semiconductors hold great promise in future display technology, owing to their high mobility, good transparency, and scalability [4,5,6,7]. Commercial metal oxides are grown via physical vapor deposition technologies, but solution-based approaches have been attracting particular attention recently [5,8,9,10,11]. Compared with conventional vacuum-based technologies, the solution approaches have additional advantages, including cost effectiveness, atmospheric fabrication, higher throughput, and material composition that is easy to tune [12,13,14,15]. Ways to reduce defect states and improve electrical performance and stability are an urgent challenge for solution-based metal oxide TFTs [16,17]. Various approaches have been taken to solve the above challenge, such as doping, modification of components, addition of additives, and novel post-treatments [9,18,19,20]. However, electron transport properties are still hindered by these defect-prone oxides [21,22,23,24,25].

**Figure 1:**
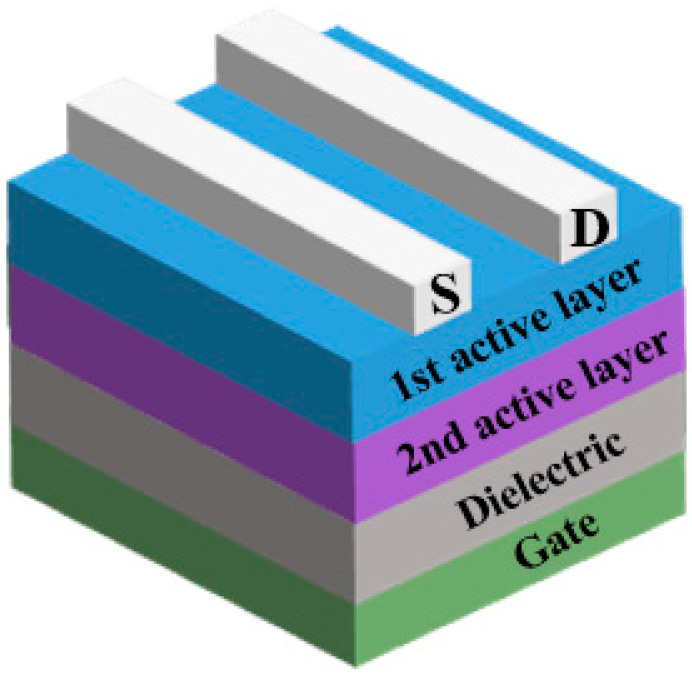
Cross-sectional view of the heterojunction oxide thin-film transistors (TFTs).

A notable strategy has been recently developed to enhance the electrical performance of solution-derived oxide TFTs by utilizing heterojunction channels [26]. The schematic of the heterojunction oxide TFTs is demonstrated in Figure 1. It is revealed that heterostructures could modulate electrical performance by taking advantage of both the front channel (providing high mobility) and the back channel (maintaining low off current) [27,28]. More importantly, some recent studies argue that the presence of a 2D electron gas system formed at the carefully engineered oxide heterointerface can greatly improve device mobility [24,29,30,31,32,33]. In this review, we summarize the recent progress of solution-processed heterostructure oxide TFTs. The heterojunction channel strategy could address the shortcomings of single-layer devices, providing a new route for future TFT technology development [34,35,36].

## 2. Heterojunction Oxide TFTs

### 2.1. Vacuum-Processed Heterojunction Oxide TFTs

Before reviewing solution-processed heterojunction oxide TFTs, we would like to make a short introduction on vacuum-based heterojunction oxide devices. In 2008, Kim et al. produced InSnO/GaInZnO (ITO/GIZO) heterojunction TFTs by magnetron sputtering, with a high mobility of 104 cm^2^/Vs, a suitable threshold voltage (*V_th_*) of 0.5 V, and a low V_th_ shift of 0.75 V for 4 h under 10 V bias voltage [37]. They found that the lower layer of highly conductive oxides could provide high mobility for the TFTs, while the upper layer of oxides with lower carrier concentration could adjust the threshold voltage. This new structure provides a new way to adjust the performance of TFTs. Subsequently, a number of scientists have studied and produced various excellent heterojunction oxide TFTs. In 2014, Chen et al. prepared InSnO/SnZnO (ITO/TZO) TFT on a glass substrate by taking advantage of ITO’s higher carrier concentration and TZO’s ability to control the charge conductance, and they obtained a high mobility of 105 cm^2^/Vs [38]. In 2016, Cong et al. built quasi-double-channel (QDC) AlSnZnO (ATZO) TFTs with a superior mobility of 108 cm^2^/Vs and an on/off ratio of 10^9^ [36]. In 2019, He et al. prepared InGaZnO/In_2_O_3_ (IGZO/In_2_O_3_) TFTs by magnetron sputtering at room temperature, exhibiting high mobility (64.4 cm^2^/Vs) and high on/off ratio (10^7^), with large enhancement compared with single-layer IGZO and In_2_O_3_ TFTs [39]. They attributed this improvement to the defect self-compensation mechanism between the two layers. In 2019, Furuta et al. prepared IGZO/IGZO TFTs with a mobility of 24.7 cm^2^/Vs and an on/off ratio of 10^7^ [40]. Table 1 summarizes the recent progress in vacuum-processed heterojunction oxide TFTs. It can be observed that heterojunction oxide TFTs show excellent electrical properties, which are much better than those of the traditional single-layer device.

### 2.2. Solution-Processed Heterojunction Oxide TFTs

**Figure 2:**
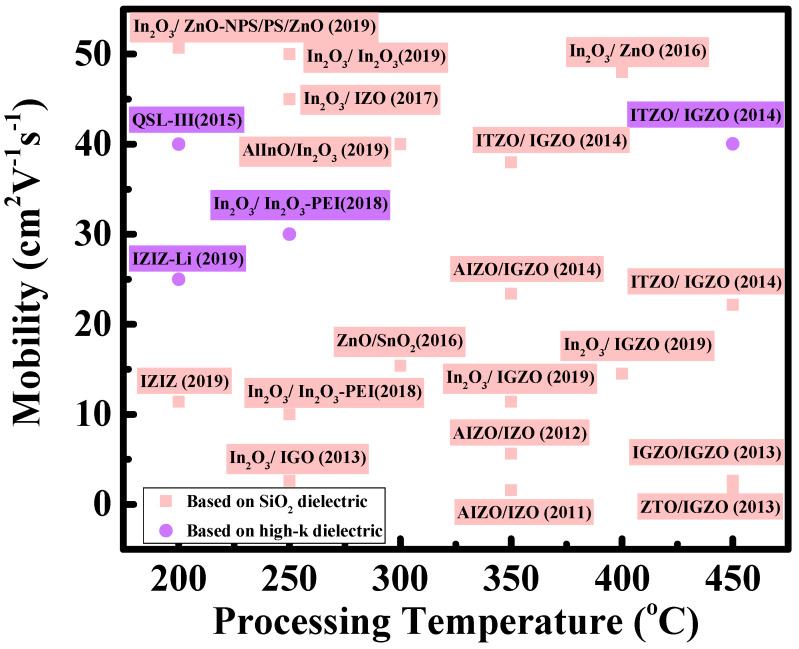
Mobility vs. processing temperature for solution-processed heterojunction oxide TFTs from Table 2 or high-k dielectrics. (QSL-III denotes In_2_O_3_/Ga_2_O_3_/ZnO/Ga_2_O_3_/In_2_O_3_; PEI-In_2_O_3_ denotes polyethylenimine-doped In_2_O_3_; IZIZ denotes In_2_O_3_/ZnO/In_2_O_3_/ZnO; Li-IZIZ denotes In_2_O_3_/ZnO-Li/In_2_O_3_/ZnO-Li.)

Compared with conventional vapor-based techniques, solution processing (such as spin-coating, spraying, and printing) allows for the design and fabrication of novel oxide TFTs in a low-cost and straightforward fashion [43,44,45]. Many researchers have begun to study solution-grown heterojunction oxide TFTs. Table 2 and Figure 2 summarize the recent advances in solution-processed heterojunction oxide TFTs. For heterojunction oxide TFTs, we discuss two of the most prominent advantages, namely electrical-property modulation and mobility enhancement by forming 2D electron gas.

#### 2.2.1. Electrical-Property Modulation

As a product of structural engineering technology, heterojunction oxide TFTs can take advantage of the excellent electrical properties of each layer [30,37]. As the front-channel layer has good conductivity, it can provide higher carrier concentration, thus forming maximum charge accumulation and finally achieving high mobility [10,41,42]. The carrier concentration in the back-channel layer is much lower than that of the front-channel layer, which leads to the difference in electron activation energy between the conduction band minimum and the Fermi energy level, forming an energy barrier at the interface [51]. Due to the existence of an energy barrier, the back-channel layer can effectively control the electron flow, thus reducing off current (*I_off_*) and adjusting threshold voltage (*V_th_*) [46]. A series of representative papers show that heterojunction TFTs can achieve both high mobility and *I_off_* by selecting suitable front- and back-channel materials.

In 2012, Jeong et al. adjusted the carrier concentration of the AlInZnO/InZnO (AIZO/IZO) interface and barrier height by changing the ratio of In/Zn in the IZO layer and the thickness of the IZO layer. The IZO layer could provide an enhanced mobility for the device due to its high electron concentration. Compared with the IZO layer, the AIZO layer had a larger *E_C_–E_F_* due to its lower carrier concentration, forming an energy barrier at the interface and reducing *I_off_*. As the thickness of the conductive IZO layer decreased, the AIZO/IZO TFTs V_th_ shifted positive, and *I_off_* decreased from 10^−8^ to 10^−11^ A. With a 12-nm-thick IZO layer, they obtained a device mobility of 5.63 cm^2^/Vs and an on/off ratio of 10^6^ [46]. For similar reports, refer to Kim et al. ZnSnO/InGaZnO (ZTO/IGZO), Yu et al. In_2_O_3_/InGaO (In_2_O_3_/IGO), Kim et al. InGaZnO/InGaZnO (IGZO/IGZO), Seo et al. AIZO/IZO, and Lee et al. In_2_O_3_/In_2_O_3_ (amorphous In_2_O_3_ and polycrystalline In_2_O_3_) [47,48,49,50].

**Figure 3:**
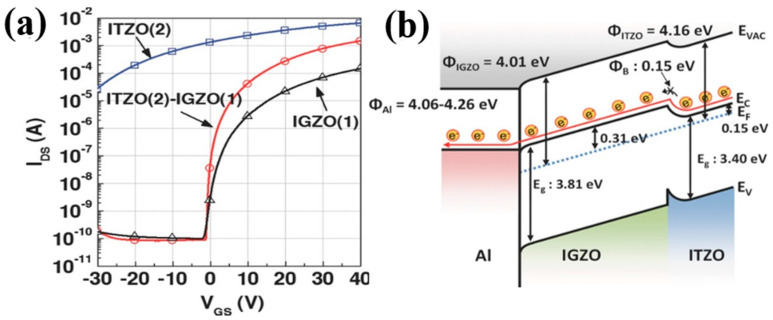
(**a**) Transfer characteristics of InGaZnO (IGZO), InSnZnO (ITZO), and ITZO/IGZO TFTs. (**b**) Energy band diagram of ITZO/IGZO. Reproduced with permission [51]. Copyright 2014, Wiley-VCH.

Rim et al. boosted up the mobility of solution-processed oxide TFTs using an extremely thin layer of conductive InSnZnO (ITZO) inserted between the dielectric layer and the InGaZnO (IGZO) active layer [51]. The ITZO/IGZO TFTs have a high mobility (22.16 cm^2^/Vs) and an excellent on/off current ratio (10^7^). As shown in Figure 3a, the mobility of ITZO/IGZO is over ten times higher than that of single-layer IGZO (from 1.56 to 22.16 cm^2^/Vs). At the front channel of ITZO, Sn^4+^ replaced In^3+^ to provide additional electrons to increase electron concentration, forming a highly conductive channel and providing high mobility for devices. Moreover, a barrier height (0.15 eV) between IGZO and ITZO (Figure 3b) could effectively modulate off current and threshold voltage. Nadarajah et al. (2015) also tried solution-processed ITZO/IGZO TFTs, showing a mobility of ~30 cm^2^/Vs and an *I_on_/I_off_* of 10^6^ [59].

**Figure 4:**
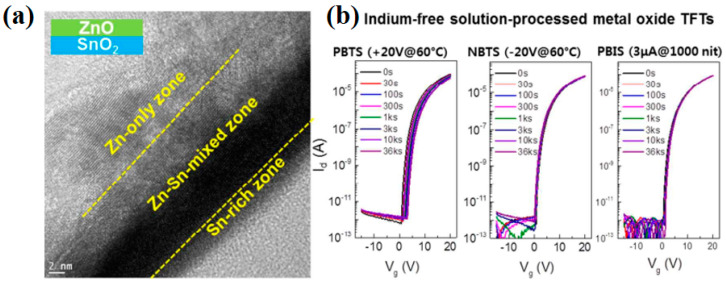
(**a**) Transmission electron microscope (TEM) of ZnO/SnO_2_ heterojunction thin film. (**b**) Transfer curve evolutions for ZnO/SnO_2_ heterojunction TFTs against a variety of bias stresses. Reproduced with permission [53]. Copyright 2016, Royal Society of Chemistry.

Nam et al. prepared high-performance solution-processed indium-free ZnO/SnO_2_ TFTs at 300 °C by UV annealing [53]. The ZnO/SnO_2_ TFTs exhibited a mobility of 15.4 cm^2^/Vs, an outstanding on/off ratio of 10^8^, and superior bias stability. As shown in Figure 4a, ZnO/SnO_2_ bilayers are composed of a Zn-rich layer, a Zn-Sn mixed zone, and an Sn-rich layer. The Sn-rich channel has high conductivity and provides a path for rapid electronic transport. Meanwhile, Zn atoms can diffuse into the Zn-Sn mixing zone to reduce *I_off_* by control carrier concentration. Furthermore, due to the suppression of oxygen vacancy (*V_o_*) defects in the bilayer film, the ZnO/SnO_2_ TFTs exhibited remarkable bias-stress stability (Figure 4b).

#### 2.2.2. Mobility Enhancement by Forming 2D Electron Gas

In addition to electrical-performance modulation, some recent studies suggest that well-designed heterojunction oxides could greatly boost mobility by forming 2D electron gas at the interface [52]. Through careful interface engineering, electron transfer and confinement at the heterointerface can occur because of a large conduction band offset between the two layers, resulting in the formation of 2D electron gas in the interface [55]. The formation of 2D electron gas enables the realization of TFTs with mobilities close to the theoretical limit set by phonon scattering in the absence of impurity scattering [30]. In this situation, the mobility of the heterojunction device is often several times or even ten times higher than that of the single-layer device. Additionally, the enhanced electron mobility is accompanied by a marked change in the charge transport mechanism. Through fitting the transfer curves and analyzing the temperature dependence of mobility, it was revealed that heterojunction TFTs exhibited band-like electron transport, while the single-layer device showed trap-limited conduction [55]. It should be mentioned that the transfer curve of the heterojunction TFTs shifted to the negative direction compared with the single-layer device, due to the formation of 2D electron gas.

**Figure 5:**
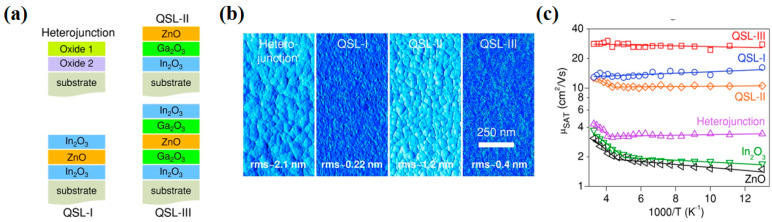
(**a**) Thin-film schematics of heterojunction quasi-superlattices (QSLs) consisting of In_2_O_3_/ZnO/In_2_O_3_ (QSL-I), In_2_O_3_/Ga_2_O_3_/ZnO (QSL-II), and In_2_O_3_/Ga_2_O_3_/ZnO/Ga_2_O_3_/In_2_O_3_ (QSL-III). (**b**) Atomic force microscope (AFM) surface phase images of the different layered structures. (**c**) Arrhenius plots of the temperature dependence of the electron mobility for different layered structures. Reproduced with permission [52]. Copyright 2015, Wiley-VCH.

Faber et al. demonstrated In_2_O_3_/ZnO TFTs with unprecedented electron mobility grown from the solution [55]. The mobility of In_2_O_3_/ZnO TFTs (45 cm^2^/Vs) was 2 to 100 times greater than that of single-layer In_2_O_3_ and ZnO devices. According to X-ray photoelectron spectroscopy, optical absorption, and Kelvin probe measurements, the In_2_O_3_/ZnO interface has a large conduction band offset (0.36 eV), which makes the electron transfer from the ZnO layer to In_2_O_3_ and forms 2D electron gas. 2D electron gas greatly increases the concentration of free electrons in the In_2_O_3_ layer of the crystal. The electron transport mechanism of In_2_O_3_/ZnO TFTs was changed from trap-limited conduction to percolation conduction. This marked improvement originated from the presence of 2D electron gas formed at the atomically sharp heterointerface induced by the large conduction band offset between In_2_O_3_ and ZnO. The In_2_O_3_/ZnO TFTs developed in this work not only surpassed the performance of single-layer In_2_O_3_ and ZnO TFTs but also compared favorably to state-of-the-art vacuum-processed devices. Lin et al. used solution-grown In_2_O_3_, Ga_2_O_3_, and ZnO to construct heterojunction and quasi-superlattice (QSL) TFTs (Figure 5a) [52]. By carefully optimizing the structure, QSLs with smooth interfaces and surfaces could be realized (Figure 5b). As shown in Figure 5c, it was proved that single-layer metal oxide TFTs were dominated by trap-limited conduction (TLC), while QSL-I/III were dominated by percolation conduction (PC). The change of electron transport mode led to a great increase in electron mobility (from 4 to 30 cm^2^/Vs).

Later studies showed that layer configuration and annealing temperature greatly affect heterojunction device performance. Khim explored the effect of layer configuration on electron transport in heterojunction transistors composed of ZnO and In_2_O_3_ [57]. They found that depositing In_2_O_3_ first followed by ZnO resulted in a smooth interface, while reversing the layer order yielded poor interface roughness. Tetzner et al. studied the influence of annealing temperature on morphology, chemical state, and electrical performance of solution-based heterostructure In_2_O_3_/ZnO TFTs [54]. It was found that the annealing temperature changed surface roughness and atomic diffusion at the interface of In_2_O_3_/ZnO. At the annealing temperature of 400 °C, the In_2_O_3_/ZnO TFTs showed an optimized mobility of 48 cm^2^/Vs and an on/off current ratio of ~10^4^.

**Figure 6:**
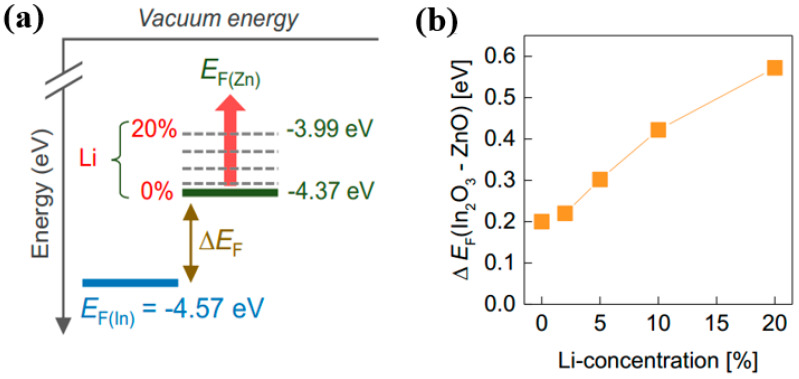
(**a**) Evolution of the Fermi energy level in In_2_O_3_ and ZnO layers as a function of Li concentration. (**b**) Schematic energy band diagram illustrating the shift of E_F(Zn)_ to higher energies with increasing Li concentration in relation to E_F(In)_. Reproduced with permission [29]. Copyright 2017, Wiley-VCH.

The large conduction band offset between the two layers is one of the key points to induce 2D electron gas for mobility improvement. A doping strategy has been adopted to enlarge the conduction band offset between the two layers. Khim and co-workers reported the controlled growth of In_2_O_3_/Li-ZnO TFTs by modulation doping [29]. It was revealed that Li addition in ZnO led to n-type doping and allowed for the accurate tuning of its Fermi energy. Therefore, doping of Li could precisely regulate ΔE_F_ between In_2_O_3_ and Li-ZnO and change the conduction band offset between the two layers (Figure 6). When the doping amount of Li was 20%, the mobility of In_2_O_3_/Li-ZnO heterojunction TFTs reached the maximum value of 11.4 cm^2^/Vs and the on/off current ratio of ~10^5^. Chen et al. demonstrated high performance In_2_O_3_/In_2_O_3_:polyethylenimine (PEI) heterostructure TFTs [56]. The 2D electron gas was achieved by creating a band offset between In_2_O_3_ and In_2_O_3_:PEI via work function tuning of the PEI-doping ratio. The resulting device exhibited a mobility of 10 cm^2^/Vs on SiO_2_ gate dielectric. Similarly, Liu et al. took In_2_O_3_ as the front channel and combined it with the back-channel AlInO to construct heterojunction transistors. By adjusting the thickness of AlInO and the doping amount of Al, AlInO (30%)/In_2_O_3_ heterostructure TFTs with a high mobility of 40 cm^2^/Vs, a threshold slope of 0.7 V/dec, and an on/off ratio of 10^7^ could be realized [58].

## 3. Conclusions and Outlooks

Great progress has been made in the past few years in solution-processed heterojunction oxide TFTs. A detailed review of this topic has been presented, with special attention on the latest developments over the past 5 years. It was revealed that heterojunction channels could overcome the disadvantages of single-layer structures. By using this novel strategy, solution-based oxide transistors with high mobility (~50 cm^2^/Vs) and operational stability could be realized, competing with or even surpassing vacuum-grown counterparts.

In terms of future research directions, several key issues need to be addressed. First, the reported high mobility heterojunction oxide TFTs often suffered from negative threshold voltages, high off-state currents, or poor stability, which have a negative impact on commercial applications. The negative threshold voltage and high off-state current of heterojunction oxide TFTs are closely related to the formation of 2D electron gas. Lee et al. constructed corrugated structure ITZO/IGZO TFTs with both high mobility (51 cm^2^/Vs) and low off-state current [30]. The thin ITZO/IGZO portion increased the overall resistivity of the current path, effectively reducing the off-state current. The thick ITZO/IGZO could provide free electrons to form a high-speed electronic channel, highly improving the electron mobility. By using this new corrugated heterojunction, solution-based oxide TFTs with high mobility and low off current could be realized. Unfortunately, this could increase the complexity of the process. Lin et al. reported solution-processed ZnO/ZnO-NPs/PS/In_2_O_3_ multilayer TFTs with high electron mobility (50 cm^2^/Vs) and prolonged operational stability [31]. Insertion of the ozone-treated polystyrene interlayer could passivate electron traps in the channel, leading to high mobility and excellent operational stability. However, continued work should be carried out. It should be mentioned that well-optimized single-layer vacuum-based metal oxide TFTs show high performance and stability. Secondly, previous studies argued that the mobility enhancement is attributed to the 2D electron gas that formed at the heterogeneous interface. However, the traditional 2D electron gas usually exists in high-quality epitaxial heterojunction systems (such as AlGaAs/GaAs heterojunctions). The oxide thin films prepared by the solution method are usually polycrystalline or amorphous, and the 2D electron gas formation mechanism is still unclear. Thirdly, the carrier transport properties in heterojunction oxide need further investigation. A deep understanding of the heterojunction electron transport can promote better design of high-performance heterojunction oxide devices. Fourthly, the previous research mainly focused on the In_2_O_3_/ZnO system; it is necessary to extend this to other multicomponent oxide semiconductor heterostructures (such as the IGZO system) for further device performance improvement. We believe that by addressing the issues presented above, solution-processed heterojunction oxide TFTs will show great promise in future large-area and high-performance electronics.

## Figures and Tables

**Table 1:** Recent advances in vacuum-processed heterojunction oxide TFTs and representative single-layer devices.

Channel	Mobility (cm^2^·V^−1^·s^−1^)	*Ion/Ioff*	Subthreshold Swing	Dielectric	Year	Reference
ITO/GIZO	104	10^8^	0.25	PECVD SiO_2_	2008	[37]
ZTO/ITO	52	10^8^	-	PECVD SiO_2_	2010	[7]
IZO/IGZO	30	10^8^	-	PECVD SiO_2_	2010	[11]
H_x_IZO/H_y_IZO	15	10^10^	-	Thermal SiO_2_	2010	[3]
IGZO/GZO	10	10^7^	0.93	Thermal SiO_2_	2011	[6]
IGZO/ZIO	18	10^10^	-	PECVD SiO_2_	2011	[10]
ZTO/ITO	43	10^7^	0.18	PECVD SiO_2_	2011	[41]
HIZO/IZO	41.4	10^7^	1.45	Thermal SiO_2_	2011	[42]
IZO/GIZO	48	10^10^	-	PECVD SiO_2_	2012	[27]
HIZO/IZO	48	10^7^	0.28	PECVD SiO_2_	2012	[13]
IGZO/IGZO:Ti	63	10^6^	0.73	HfO_2_	2014	[22]
ZTO/IZO	32	10^8^	0.20	PECVD SiO_2_	2014	[23]
ITO/TZO	105	10^7^	0.33	PECVD SiO_2_	2014	[38]
In_2_O_3_/IZO	38	10^9^	0.12	ZrO_2_	2014	[24]
High-O-IGZO/Low-O-IGZO	60	10^8^	0.2	Thermal SiO_2_	2014	[34]
IZO/AZTO	53.2	10^10^	0.15	PECVD SiO_2_	2016	[12]
ZnO-H/ZnO	43	10^8^	0.13	Thermal SiO_2_	2016	[35]
L-AZTO/H-ATZO	108	10^9^	0.15	PECVD SiO_2_	2016	[36]
IG_X_O/IG_Y_O	53.2	10^7^	0.19	PECVD SiO_2_	2017	[25]
AIZTO/IZO	53	10^10^	0.15	PECVD SiO_2_	2018	[32]
In_2_O_3_/IGZO	64.4	10^7^	0.20	Thermal SiO_2_	2019	[39]
In_2_O_3_/IGZO	67.5	10^7^	0.08	HfO_2_	2019	[39]
In_2_O_3_/IGZO	79.1	10^7^	0.09	Si_3_N_4_	2019	[39]
ZnO(DEZ+O_3_)/ZnO(DEZ+H_2_O)	31.1	10^7^	0.21	Al_2_O_3_	2019	[33]
IGZO/IGZO	24.7	10^7^	0.1	Thermal SiO_2_	2019	[40]
SnO_2_	35.4	10^7^	-	Thermal SiO_2_	2015	[43]
ZnO	20	10^5^	0.38	TiO_2_/Al_2_O_3_	2015	[44]

**Table 2:** Recent advances in solution-processed heterojunction oxide TFTs and representative single-layer devices.

Channel	Processing Temperature (°C)	Mobility (cm^2^·V^−1^·s^−1^)	*I_on_/I_off_*	Subthreshold Swing	Dielectric	Year	Reference
AIZO/IZO	350	1.57	10^7^	0.59	SiO_2_	2011	[26]
AIZO/IZO	350	5.62	10^6^	0.53	SiO_2_	2012	[46]
IGZO/IGZO	450	2.4	10^7^	0.69	SiO_2_	2013	[47]
ZTO/IGZO	450	2.09	10^7^	0.49	SiO_2_	2013	[48]
In_2_O_3_/IGO	250	2.6	10^8^	-	SiO_2_	2013	[49]
AIZO/IZO	350	23.4	10^7^	0.27	SiO_2_	2014	[50]
ITZO/IGZO	450	22.16	10^7^	0.51	SiO_2_	2014	[51]
ITZO/IGZO	450	40.03	10^5^	0.12	ZrO_2_	2014	[51]
QSL-III ^1^	200	40	10^4^	0.27	AlO_x_/ZrO_x_	2015	[52]
ZnO/SnO_2_	300	15.4	10^7^	-	SiO_2_	2016	[53]
In_2_O_3_/ZnO	400	48	10^4^		SiO_2_	2016	[54]
In_2_O_3_/ZnO	250	45	10^7^	-	SiO_2_	2017	[55]
In_2_O_3_/Li-ZnO	350	11.4	10^5^	-	SiO_2_	2017	[29]
ITZO/IGZO	350	38	10^8^	0.41	SiO_2_	2018	[30]
In_2_O_3_/PEI-In_2_O_3_^2^	250	10	10^6^	-	SiO_2_	2018	[56]
In_2_O_3_/PEI-In_2_O_3_^2^	250	30	10^6^	-	ZrO_2_	2018	[56]
In_2_O_3_/IGZO	400	14.5	10^6^	-	SiO_2_	2019	[17]
In_2_O_3_/ZnO-NPS/PS/ZnO	200	50.7	10^6^	2.71	SiO_2_	2019	[31]
IZIZ ^3^	200	11.4	10^7^	-	SiO_2_	2019	[57]
Li-IZIZ ^4^	200	25	10^8^	-	AlO_x_/ZrO_2_	2019	[57]
AlInO/In_2_O_3_	300	40	10^7^	0.7	SiO_2_	2019	[58]
In_2_O_3_/In_2_O_3_	250	50	10^6^	-	SiO_2_	2019	[28]
IGZO	150	14	10^8^	0.17	Al_2_O_3_	2012	[14]
InSmO	350	21.5	10^8^	0.66	SiO_2_	2020	[15]

^1^ In_2_O_3_/Ga_2_O_3_/ZnO/Ga_2_O_3_/In_2_O_3_ (QSL-III). ^2^ Polyethylenimine-doped In_2_O_3_ (PEI-In_2_O_3_). ^3^ In_2_O_3_/ZnO/In_2_O_3_/ZnO (IZIZ). ^4^ In_2_O_3_/ZnO-Li/In_2_O_3_/ZnO-Li (Li-IZIZ).
